# A chemical specialty semantic network for the Unified Medical Language System

**DOI:** 10.1186/1758-2946-4-9

**Published:** 2012-05-11

**Authors:** C Paul Morrey, Yehoshua Perl, Michael Halper, Ling Chen, Huanying “Helen” Gu

**Affiliations:** 1 Department of Information Systems and Technology, Utah Valley University, 800 West University Parkway, Orem, UT 84058, USA; 2 Structural Analysis of Biomedical Ontologies Center, Department of Computer Science, New Jersey Institute of Technology, University Heights, Newark, NJ 07102, USA; 3 Information Technology Program, New Jersey Institute of Technology, University Heights, Newark, NJ 07102, USA; 4 Department of Science, Borough of Manhattan Community College, City University of New York, 199 Chambers Street, New York, NY 10007, USA; 5 Department of Computer Science, New York Institute of Technology, 1855 Broadway, New York, NY 10023, USA

**Keywords:** Unified Medical Language System, Vocabulary, Controlled, Semantics, Models, Chemical, Chemical characterization, Chemical Entities of Biological Interest, Semantic Network

## Abstract

**Background:**

Terms representing chemical concepts found the Unified Medical Language System (UMLS) are used to derive an expanded semantic network with mutually exclusive semantic types. The UMLS Semantic Network (SN) is composed of a collection of broad categories called semantic types (STs) that are assigned to concepts. Within the UMLS’s coverage of the chemical domain, we find a great deal of concepts being assigned more than one ST. This leads to the situation where the extent of a given ST may contain concepts elaborating variegated semantics.

A methodology for expanding the chemical subhierarchy of the SN into a finer-grained categorization of mutually exclusive types with semantically uniform extents is presented. We call this network a *Chemical Specialty Semantic Network* (*CSSN*). A CSSN is derived automatically from the existing chemical STs and their assignments. The methodology incorporates a threshold value governing the minimum size of a type’s extent needed for inclusion in the CSSN. Thus, different CSSNs can be created by choosing different threshold values based on varying requirements.

**Results:**

A complete CSSN is derived using a threshold value of 300 and having 68 STs. It is used effectively to provide high-level categorizations for a random sample of compounds from the “Chemical Entities of Biological Interest” (ChEBI) ontology. The effect on the size of the CSSN using various threshold parameter values between one and 500 is shown.

**Conclusions:**

The methodology has several potential applications, including its use to derive a pre-coordinated guide for ST assignments to new UMLS chemical concepts, as a tool for auditing existing concepts, inter-terminology mapping, and to serve as an upper-level network for ChEBI.

## Background

The Semantic Network (SN^a^) [1-5] of the UMLS 2009AA (used for this research) [6-12] is composed of 135 broad categories called semantic types (STs) that overlay the nearly 2.1 million concepts of the Metathesaurus (META) [13-16]. A remarkable fact is that since the first version of the SN only five new STs have been added, while at the same time the number of concepts in the META—which are categorized by the STs— has grown by two orders of magnitude from about 64,000 concepts to 2.1 million concepts. Indeed, in [4], it was stated that expanding the SN’s depth of coverage was part of future plans. One can understand the need not to let the SN grow too expansive and lose its impact as a compact abstraction network. In the interim, we have seen proposals for additional STs, such as those for the coverage of genomics [17-19] and refinement and expansion in other areas [20]. There have also been suggestions about changing the structure of the SN from a tree to a directed acyclic graph [21-24]. In a workshop “The Future of the UMLS Semantic Network” (see http://mor.nlm.nih.gov/snw) organized by the NLM in 2005, various potential changes were discussed. However, no change occurred until the 2010AA release (see http://download.nlm.nih.gov/umls/kss/2010AA/README.txt) which removed three STs (**Invertebrate**^b^**Rickettsia or Chlamydia**, and **Alga**) and added one new one (**Eukaryote**).

An abstraction network, such as the SN, can be a useful aid in regards to the issues of comprehension, integration, and navigation of a large terminological system. It affords a view of the underlying concept repository that is compact yet gives an idea of the gestalt of the content. Abstraction networks may have different levels of granularity even when they cover the same area of the terminology. In addition to the SN, abstraction networks for the UMLS include a metaschema [25], Semantic Groups [26], and the Refined Semantic Network [24].

Within the context of the UMLS, we find an extensive treatment of chemical concepts. In that subdomain, there are many concepts that are assigned more than one ST. Indeed, the definition of the ST **Chemical** states: “…Chemicals are viewed from two distinct perspectives in the network, functionally and structurally. Almost every chemical concept is assigned at least two types, generally one from the structure hierarchy and at least one from the function hierarchy.” (See http://semanticnetwork.nlm.nih.gov/Download/RelationalFiles/SRDEF.) A disadvantage of this is that it leads to the situation where the extent of a given ST may contain concepts elaborating variegated semantics. For example, one concept, say, *C*_1_ assigned the ST **X** may simultaneously be assigned **Y**, while another *C*_2_ assigned **X** may be assigned **Z** at the same time. Thus, in the extent of **X**, we find *C*_1_ which is an **X** and a **Y**, and *C*_2_ which is an **X** and a **Z**. This arrangement cannot be ascertained from the level of the SN, which degrades its utility as an abstraction network because the organization of collections of concepts into groups that share the same ST assignment results in groups of semantically inconsistent concepts. Indeed, in [27] we observed that using the current SN a conjugate chemical is not distinguished from a complex chemical.

In this paper, we present a methodology for expanding the portion of the SN focused on chemicals with additional types in order to acquire a finer-grained *Chemical Specialty Semantic Network* (*CSSN*). The CSSN will comprise types that are in fact disjoint, meaning all concepts will be assigned one and only one type. This arrangement better supports user comprehension and auditing. The derivation of a CSSN utilizes techniques previously developed for the Refined Semantic Network (RSN) [21,22], which promotes multiple ST combinations to the level of first-class types.

Let us emphasize that we are not necessarily proposing to expand the SN itself, but are allowing portions of it to be expanded according to users’ and applications’ needs. In fact, the work completely leverages the existing SN framework and the current assignments of STs to concepts. That is, any new type (called an *intersection semantic type* (*IST*)) appearing in a CSSN is derived strictly from existing STs based on the underlying assignment of those types to concepts in the META. No effort is spent on manually adding new knowledge to the UMLS. The ISTs are named in meaningful ways that express the type combinations. Moreover, the user is given control of a threshold parameter governing the minimum number of concepts required to be assigned an IST in order for it to qualify for inclusion in a CSSN. Choosing this threshold parameter allows for the creation of various CSSNs, each exhibiting a different depth of display.

We demonstrate the novel use of a CSSN as an upper-level compact semantic network for an ontology of chemicals, the Chemical Entities of Biological Interest (ChEBI) [28], an ontology for biochemistry of the Open Biological and Biomedical Ontologies (OBO) Foundry. For our demonstration, we describe our use of the CSSN to categorize a random sample of ChEBI chemical compounds. The categorization of these compounds into CSSN types has potential application for mapping data sources—especially if one of the sources is a UMLS source vocabulary. The need for semantic models in chemistry is described in [29]. The CSSN is an effort towards building a resource with application to ontologies, terminology mapping, and natural language processing.

The Refined Semantic Network (RSN) [21,22] was introduced as a finer-grained UMLS categorization network that exhibits so-called semantically uniform type extents. To distinguish between the STs of the SN and the types of the RSN we call the RSN types *refined semantic types* (*RST*s). These RSTs, derived from the SN and the META, consist of two kinds: *pure semantic types* (*PSTs*), each corresponding to a specific ST in the SN, and *intersection semantic types* (*ISTs*). A PST assignment is used for a concept that is assigned only one ST. For example, *Neuromuscular transmission drug* (C0357560) is assigned just **Pharmacologic Substance** and therefore retains this assignment in the RSN with an assignment to the PST of the same name.

The ISTs are reifications of combinations of multiple ST-assignments. Specifically, if a concept *C* is assigned two STs, say, **A** and **B**, then an IST appears in the RSN representing the assignment of both **A** and **B**. This IST is named **A** ∩ **B**, where “∩” denotes set intersection. Instead of assigning *C* both **A** and **B**, it is assigned only the type **A** ∩ **B**. For example, *Baclofen*^c^ (C0004609) is assigned both **Amino Acid, Peptide, or Protein** and **Pharmacologic Substance**. Therefore, in the RSN it is assigned only the IST **Amino Acid, Peptide, or Protein** ∩ **Pharmacologic Substance**.

The construction of ISTs via the combination of multiple STs depends on the existing assignment of STs to concepts, following the UMLS’s practice of post-coordination for type assignment. Overall, the extents of the RSTs are disjoint and form a partition of the META. The concepts in the extent of a particular type in the RSN all share the same original ST assignments. Due to this, we say the extents of the RSTs are semantically uniform, a property that is not true for the extents of the STs. For example, all the concepts assigned exactly the IST **Pharmacologic Substance** ∩ **Lipid** are lipids used pharmacologically, while all the concepts assigned the PST **Lipid** are just lipids. In contrast, the extent of the ST **Lipid** from the SN is not semantically uniform since some concepts are just lipids while others are also used pharmacologically.

One application of the RSN has been in auditing the UMLS. Our experience is that ISTs with small extents (six or fewer concepts) often contain concepts with erroneous ST assignments [21,22,27,30]. For example, in a sample of 59 chemical concepts from ISTs assigned to just one concept, 20 were found to have incorrect ST assignments [30]. As an illustration, the concept *Metaltite* (C1700474) was the only one in the UMLS 2006AB assigned both **Nucleic Acid, Nucleoside, or Nucleotide** and **Biomedical or Dental Material**. Upon analysis, it was found that this concept represents Metaltite®, a primer used to improve adhesion between resins and precious metals. The assignment of **Nucleic Acid, Nucleoside, or Nucleotide** was thus in error. The NLM corrected this in the UMLS 2007AB by changing the ST assignment to **Organic Chemical**. As a result of correcting such ST assignments, the corresponding 20 chemical ISTs disappeared from the RSN. In another audit sample of ten chemical ISTs assigned to just two chemical concepts each, 16 of the 20 concepts were found to have ST assignment errors, and nine of these ten chemical ISTs were slated for removal [27]. More recently, we have reviewed concepts of chemical intersections with six or fewer concepts in the UMLS 2009AB and found that 73 of the existing 151 ISTs only exist because of current inconsistent ST assignments given the concepts of their extents. As these inconsistencies are resolved by the UMLS editors, these 73 small ISTs are expected to disappear from future versions of the RSN. Our CSSN can be used in the same way as the RSN to determine partitions of concepts in the META that merit the review of an auditor by choosing a threshold value of one and selecting ISTs of extents of up to six concepts [30,31].

The ChEBI ontology is used by a variety of computer systems and projects for tasks such as cataloging chemical transformations [32], identification of small molecules by matching electron ionization-mass spectra [33], and the development of a chemical dictionary for text mining [34]. The ChEBI is independent of the UMLS in organization, structure, and source data.

## Experimental

In this section, we first describe the derivation of the types for a Chemical Specialty Semantic Network (CSSN) from the RSN, followed by a description of the use of the threshold value to exclude certain types. After that, we present our means for dealing with type assignment for concepts whose original types have gone away. Naming conventions are then presented for the types of a CSSN, and the details of its hierarchical configuration are given. Finally, our application of a CSSN to ChEBI is described.

### Deriving the types of the chemical specialty semantic network

The Chemical Specialty Semantic Network (CSSN) was extracted from the RSN in the following manner. The CSSN included every RST in the RSN that was derived from **Chemical** or one of its descendants. We will call this group of RSTs the Chemical RSTs. According to the definition of the RSN, this implies that for any combination of several Chemical STs for which there were concepts assigned exactly to this combination, there existed an IST that was potentially included in the CSSN. For example, because in the META there were 490 concepts assigned exactly **Amino Acid, Peptide, or Protein** and **Antibiotic**, the CSSN included an IST **Amino, Acid, Peptide, or Protein** ∩ **Antibiotic**. Out of the 381 total ISTs in the RSN for the UMLS 2009AA release, 348 consisted of combinations of STs in which at least one ST was in the subtree of **Chemical**.

As noted, multiple ST-assignments naturally occurred for the STs in the subtree rooted at **Chemical** due to the typical combination of structural and functional dimensions mentioned in its definition. For example, the extent of IST **Organic Chemical** ∩ **Pharmacologic Substance** had 76,832 concepts. Furthermore, many chemical substances displayed multiple functional aspects, including the 1,940 concepts that were assigned **Amino Acid, Peptide, or Protein** ∩ **Pharmacologic Substance** ∩ **Immunologic Factor**. Among the 100 ISTs with the largest extents in the UMLS 2009AA, only eight did not pertain to chemicals.

In this study we used the intersection semantic types which we introduced in our previous research [22] about the RSN. However, we realized that the large number of the refined semantic types in the RSN is preventing the use of intersection semantic types as first-class types. By concentrating this study on the chemical concepts in the META and the chemical semantic types, the emerging CSSN abstraction network is of a smaller magnitude which will enable its adoption in a practical way for the chemical research community, by providing a more refined categorization. Another advantage is that the extents of the types of the CSSN provide semantically uniform categorization of all concepts, which is not true for the SN.

The few concepts with ST assignments to both chemical and non-chemical STs were included when materializing the CSSN by only taking into account the chemical STs. The non-chemical STs were ignored. For example, the concept *Soap* (C0037392) was assigned two STs in the META, one a chemical and one a non-chemical. For the CSSN, we consider *Soap* a chemical with the ST **Lipid** and ignore the fact that in the META it is also assigned the non-chemical type **Manufactured Object**. The largest two groups of such concepts are combinations of ST **Food** with a chemical ST of **Carbohydrate** or **Lipid**, such as *Dietary Sucrose* (C0376597), and *Nut Oil* (C1518477).

## Methods

### Use of a threshold value to determine inclusion of an intersection semantic type

The extent of a given IST can be quite small, in fact, as small as one concept. It may be argued that a collection of concepts of a small size should not qualify as a type unto itself since a type is a broad category. To address this issue, we allowed for the specification of a threshold value *N* on IST extent sizes for qualification as a type. If an IST’s extent size was below *N*, then it was omitted from the CSSN. As a consequence, some chemical concepts in the META lost IST-coverage. (This will be discussed further in Section Type Coverage for Concepts in ISTs Falling Below the Threshold Value.) Varying *N* enabled the generation of a CSSN tailored to the needs of a specific application. It should be kept in mind that the choice of *N* is a tradeoff between the CSSN’s size and the percentage of concepts it covers. For the purpose of auditing, a value of *N* = 1 would certainly be warranted as many errors occur in concepts assigned ISTs that have very small extents [21,22,27,30,31].

### Type coverage for concepts in ISTs falling below the threshold value

When a threshold value *N* > 1 is used to generate a CSSN, there will be some concepts that lose IST-coverage. This may be an acceptable tradeoff for some applications. However, for applications in which the loss of type coverage of concepts is unacceptable, the best solution is to assign the concept the IST that has the most closely related semantics. This can be taken to be the more general or “ancestor” types of the IST that is being dropped. For example, the concept *Adrenal cortex agent* (C1445697) was the only concept in the UMLS 2009AA that was assigned exactly the three STs **Organic Chemical**, **Hormone**, and **Immunologic Factor**. Consequently, when *N* > 1, the IST **Organic Chemical** ∩ **Hormone** ∩ **Immunologic Factor** did not appear in the resulting CSSN. However, the two ancestor ISTs **Organic Chemical** ∩ **Hormone** and **Organic Chemical** ∩ **Immunologic Factor** existed in the CSSN with extent sizes of 62 and 136, respectively. Either of these ISTs provided better coverage for *Adrenal cortex agent* than any other type in the CSSN.

We proposed two approaches to resolve this issue. In the first, we simply added the concept to the IST with the larger extent size. For example, *Adrenal cortex agent* should be assigned the **Organic Chemical** ∩ **Immunologic Factor** because it has a larger extent size than the other possibility, **Organic Chemical** ∩ **Hormone**.

In the second approach, we gradually relaxed the specificity of types to determine a more general IST. This relaxation is applicable to ISTs derived from either multiple functional STs or multiple structural STs. Relaxation can also be applied to ISTs that represent both multiple functional and multiple structural STs. A natural grouping of chemical STs exists among types that describe structure. Another natural grouping exists among chemical semantic types that describe function. By first separating types into structural groups and functional groups and then gradually relaxing types in a group into ancestor types, the IST which maintains the most specificity available may be determined. For example, the abovementioned *Adrenal cortex agent* had two functional ST assignments, **Hormone** and **Immunologic Factor**, that shared the common parent **Biologically Active Substance**. Therefore, these two more specific functional STs could be relaxed into **Biologically Active Substance**. The single structural assignment to **Organic Chemical** is retained. Using this approach, the concept *Adrenal cortex agent* was assigned **Organic Chemical** ∩ **Biologically Active Substance**, which had an extent size of 4,151 concepts in the UMLS 2009AA.

We also demonstrate this approach in a second example using a higher threshold value and an IST with a larger extent size. Using *N* = 500, the IST **Organic Chemical** ∩ **Pharmacologic Substance** ∩ **Immunologic Factor**, which had an extent of only 249 concepts, was excluded from the CSSN. The only structural type of this IST was **Organic Chemical**. The type **Chemical Viewed Functionally** was the common ancestor of the two functional types, **Pharmacologic Substance** and **Immunologic Factor**. However, the relaxation of the types started with **Immunologic Factor** because it can be relaxed without going directly to the common ancestor. **Immunologic Factor** was relaxed to its parent type, **Biologically Active Substance**. The IST **Organic Chemical** ∩ **Pharmacologic Substance** ∩ **Biologically Active Substance** had an extent size of 507 and was therefore a valid IST in a CSSN with *N* = 500. As such, the 249 concepts assigned each of the STs **Organic Chemical**, **Pharmacologic Substance**, and **Immunologic Factor** were assigned the IST **Organic Chemical** ∩ **Pharmacologic Substance** ∩ **Biologically Active Substance** in a CSSN generated using this approach.

### Type names in the CSSN

While theoretically accurate, names of ISTs like “**A** ∩ **B**” can be cumbersome and unnatural to a user. For this reason, we went through the process of renaming them with an eye toward syntactical consistency, in the same vein as for term-name construction [35]. In general, an IST name is a concatenated list of words drawn from its STs’ names, dropping unessential words (e.g., “substance” or “chemical”). We prioritized, by putting to the right, more specific functional aspects over structural aspects and, in turn, prioritized those over more general-purpose functional aspects. For example, **Pharmacologic Steroid** is first and foremost a steroid. Example names are presented in Section Hierarchical structure of the CSSN and in Additional file 1: Table S1.

### Hierarchical structure of the CSSN

As with the RSN, a CSSN’s hierarchical structure was a directed acyclic graph rather than a tree. The derivation of its IS-As followed those of the RSN. Each PST had an IS-A relationship identical to that of its corresponding ST in the SN. On the other hand, the IS-As of an IST were derived. In fact, an IST must always have at least two parents. While it is theoretically possible that an IST could be induced by a total of four or more ST assignments, most were with respect to two or three. We denote such an IST as a *2-IST* or *3-IST*, respectively. Let us consider the IS-As of 2-ISTs and 3-ISTs. First, each 2-IST had two IS-As, one to each of the PSTs corresponding to the STs from which it was derived. As an illustration, **Pharmacologic Organic Chemical** had one IS-A to **Organic Chemical** and another to **Pharmacologic Substance**.

**Figure 1:**
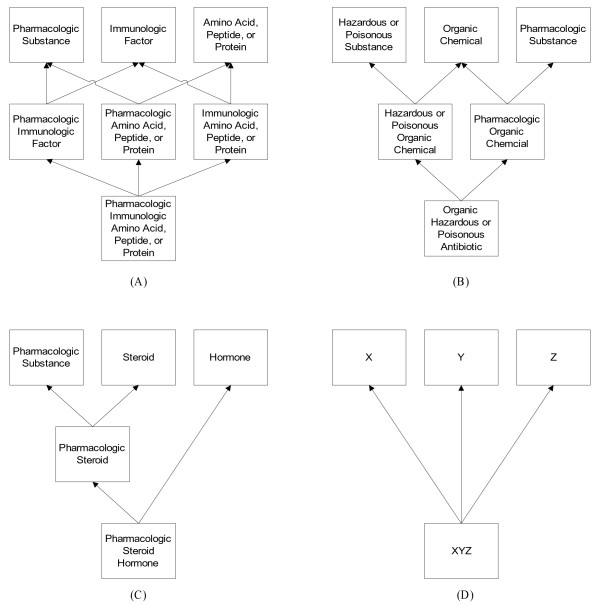
Four possible IS-A configurations for a 3-IST.

A 3-IST may have one of the four possible IS-A configurations illustrated in Figure 1. Arrows in the figure represent IS-As, and boxes represent types. Configuration (A) shows the largest 3-IST **Pharmacologic Immunologic Amino Acid, Peptide, or Protein** (with an extent of 2,087 concepts) having exactly three IS-As to the 2-ISTs **Pharmacologic Immunologic Factor**, **Pharmacologic Amino Acid Peptide or Protein**, and **Immunologic Amino Acid Peptide or Protein**. Configuration (B) shows the 3-IST **Pharmacologic Hazardous or Poisonous Organic Chemical** with a pair of IS-As to the 2-ISTs **Hazardous or Poisonous Organic Chemical** and **Pharmacologic Organic Chemical**. Configuration (C) shows the 3-IST **Pharmacologic Steroid Hormone** linked to the 2-IST **Pharmacologic Steroid** and the PST **Hormone**. Configuration (D) is theoretically possible but not actually found in the RSN derived from the UMLS 2009AA. We include it here for the sake of completeness. In that configuration, there are exactly three IS-A relationships, each directed to a PST.

### CSSN as an upper-level abstraction network for ChEBI

As a proof of concept, we applied a CSSN as an upper-level abstraction network for ChEBI in the same capacity that the SN is used for the META. Such support could be useful for mapping between the ChEBI and the UMLS source vocabularies. As a matter of fact, for those ChEBI compounds that also appeared in the UMLS, their type-assignment with respect to the CSSN was deduced automatically from their UMLS ST assignments. A random sample of ChEBI compounds was selected from version 1.50 of ChEBI.

## Results and discussion

### CSSN

A total of 53 concepts with ST assignments to both a chemical and a non-chemical ST were included in our analysis by ignoring the non-chemical ST assignment and only taking into account the assignment to the chemical ST. Examples of such concepts (*Soap*, *Dietary Sucrose*, *Nut Oil*, etc.) are described at the end of Section "Deriving the types of the chemical specialty semantic network. A CSSN was generated for a threshold value of *N* = 300, which again is applicable only to ISTs, not PSTs. This CSSN had a total of 68 types: 25 PSTs (some having less than 300 concepts) and 43 ISTs (with a minimum of 300 concepts). Table 1 shows a partial listing of this CSSN. The first column lists the RST. The second column shows the number of concepts in its extent. The third column shows the new name of the type, with appropriate hierarchical indentation utilized. PSTs appear without an asterisk. An IST is listed indented under one of its parents; 2-ISTs are preceded by one asterisk, and 3-ISTs are preceded by two asterisks. Each asterisk indicates the presence of another parent. For example, **Amino Acid, Peptide, or Protein** ∩ **Immunologic Factor** had 12,662 concepts in its extent and was renamed **Immunologic Amino Acid, Peptide, or Protein**. As noted, a 3-IST may be a child of a 2-IST. To resolve the display of the two or three parents for an IST, we located it (indented) directly under the parent ST (or 2-IST) with the higher priority, which according to our naming convention is the rightmost part in the IST’s name. For a 2-IST, denoted by “*,” the other parent can be easily identified by the leftmost word in the IST’s name. The complete listing of this CSSN can be found in Additional file 1: Table S1. A total of 62.6% (220,451 of 352,129) concepts were assigned to ISTs in the CSSN.

**Table 1:** Partial listing of the CSSN with threshold value of 300

**RST**	**# Concepts**		**Name in CSSN (hierarchically indented)**					
Chemical	26		Chemical					
Chemical Viewed Structurally	248		Chemical Viewed Structurally					
Organic Chemical	58,347			Organic Chemical				
Amino Acid, Peptide, or Protein	16,973				Amino Acid, Peptide, or Protein			
Amino Acid, Peptide, or Protein ∩ Immunologic Factor	12,662					*Immunologic Amino Acid, Peptide, or Protein		
Amino Acid, Peptide, or Protein ∩ Pharmacologic Substance	6,537					*Pharmacologic Amino Acid, Peptide, or Protein		
Amino Acid, Peptide, or Protein ∩ Pharmacologic Substance ∩ Immunologic Factor	1,940						**Pharmacologic Immunologic Amino Acid, Peptide, or Protein	
	…*and many more*…							
Chemical Viewed Functionally	174		Chemical Viewed Functionally					
Biologically Active Substance	1,026			Biologically Active Substance				
Immunologic Factor	7,181				Immunologic Factor			
Pharmacologic Substance ∩ Immunologic Factor	1,065					*Pharmacologic Immunologic Factor		
	…*and many more*…							

Table 2 shows the effect of changing the threshold value. The first column contains the threshold value (*N*). The second column shows the quantity of types that existed for a CSSN derived using the threshold value from the first column. The third column of the table shows the percentage of concepts in the META that are covered by the types available in a CSSN that is derived using the threshold value from the first column. The row with a threshold value of 300 is shaded to emphasize that the results reported in this study refer to a CSSN derived using a threshold value of 300.

**Table 2:** Effects of varying the threshold value

**Threshold value,*N***	**Number of types in CSSN**	**Percentage of coverage of concepts in META**
500	52	95.3%
300	68	97.3%
100	102	98.9%
50	123	99.4%
1	373	100.0%

There are 352,129 concepts with a chemical ST assignment in the META. Table 3 lists the chemical types in the first column. The second column shows the number of concepts with that ST assignment using the SN. Concepts with multiple ST assignments are counted more than once in the second column, once for each chemical ST assigned. The first column also corresponds to the PSTs of the CSSN. The third column shows the number of concepts assigned each PST, which are counted only once in the CSSN.

**Table 3:** Comparison of type assignments in SN and type assignments of PSTs in the CSSN

**Type name (ST in SN or PST in CSSN)**	**# of concepts assigned in SN**	**# of concepts assigned in CSSN**
Chemical	26	26
Chemical Viewed Structurally	385	248
Organic Chemical	154,400	58,347
Steroid	10,205	4,883
Eicosanoid	1,200	537
Nucleic Acid, Nucleoside, or Nucleotide	9,172	4,727
Organophosphorus Compound	2,401	919
Amino Acid, Peptide, or Protein	116,022	16,973
Carbohydrate	10,475	6,077
Lipid	6,367	3,494
Chemical Viewed Functionally	180	174
Pharmacologic Substance	122,187	14,168
Biomedical or Dental Material	4,943	3,408
Biologically Active Substance	55,049	1,026
Neuroreactive Substance or Biogenic Amine	705	20
Hormone	2,973	147
Enzyme	25,526	233
Vitamin	2,357	117
Immunologic Factor	24,305	7,181
Indicator, Reagent, or Diagnostic Aid	12,803	4,350
Hazardous or Poisonous Substance	5,750	422
Receptor	4,417	125
Antibiotic	4,554	546
Element, Ion, or Isotope	1,800	1,004
Inorganic Chemical	5,762	2,490

### ChEBI categorization

We randomly selected 200 compounds from the list of 34,055 parent compounds with names (we did not use any unnamed compounds). Of the 200 ChEBI compounds selected for examination, 42 were found in the META by manual review using both lexical and semantic matching, and their existing ST assignments were used to derive their type assignments in the CSSN. For example, the ChEBI compound 1,1-dimethylhydrazine corresponded to the UMLS concept *dimazine* (C0058187), which was assigned **Organic Chemical**. For the 151 ChEBI compounds for which synonymous concepts were not found in the META, a type assignment from the CSSN was given by one of the authors (LC), a biochemist, after reviewing the literature cited in ChEBI. Overall, 143 compounds were assigned PSTs and 49 were assigned ISTs. Only eight of the 200 compounds did not have coverage of their assigned type using the CSSN we generated. Table 4 shows the number of ChEBI compounds from the sample of 200 that we assigned to each PST or IST. Shaded rows mark ISTs of the eight compounds that do not have coverage in our CSSN because their extent size for the META is below our threshold value of 300. The percentage of coverage of the ChEBI compounds is 96.0% (192 of 200) which is very close to the 97.3% coverage of META concepts that was achieved with CSSN of the same threshold value. For the ChEBI compounds lacking a type assignment in the CSSN it would be necessary to employ one of the relaxation approaches we described in Section Type coverage for concepts in ISTs falling below the threshold value.

**Table 4:** CSSN types assigned to ChEBI sample compunds

**Pure Semantic Type or Intersection Semantic Type**	**# Compounds**
Amino Acid, Peptide, or Protein	9
Carbohydrate	3
Chemical Viewed Structurally	9
Eicosanoid	1
Element, Ion, or Isotope	6
Inorganic Chemical	9
Lipid	8
Neuroreactive Substance or Biogenic Amine	1
Nucleic Acid, Nucleoside, or Nucleotide	6
Organic Chemical	84
Organophosphorus Compound	4
Steroid	2
Biologically Active Amino Acid, Peptide, or Protein	2
Amino Acid, Peptide, or Protein Enzyme	1
Indicator, Reagent, or Diagnostic Aid Amino Acid, Peptide, or Protein	1
Neuroreactive or Biogenic Amine Amino Acid, Peptide, or Protein	1
Inorganic Biomedical or Dental Material	1
Carbohydrate Lipid	2
Pharmacologic Inorganic Chemical	2
Nucleic Acid, Nucleoside, or Nucleotide Amino Acid, Peptide, or Protein	1
Biologically Active Nucleic Acid, Nucleoside, or Nucleotide	1
Nucleic Acid, Nucleoside, or Nucleotide Carbohydrate	1
Organic Antibiotic	7
Biologically Active Organic Chemical	6
Hazardous or Poisonous Organic Chemical	4
Organic Indicator, Reagent, or Diagnostic Aid	2
Pharmacologic Organic Chemical	20
Pharmacologic Organic Hormone	1
Organic Vitamin	1
Organophosphorus Indicator, Reagent, or Diagnostic Aid	1
Hazardous or Poisonous Inorganic Chemical	1

The CSSN (e.g., with *N* = 300) promotes common combinations of chemical ST assignments to the level of types. This is expressed by naming them according to the combination semantics they elaborate. Our previous work in auditing the UMLS shows that concepts assigned ISTs with extents of six or fewer concepts tend to indicate inconsistent ST assignments [21,22,27,30,31]. Over time, the correction of these inconsistent assignments in the UMLS will reduce the number of ISTs with extents below the threshold value in a derived CSSN. In turn, this will raise the percentage of coverage for the various thresholds: the corresponding concepts will move to other, potentially larger, ISTs.

A UMLS chemical concept drawn from several source vocabularies may very well be assigned various STs by different UMLS editors—not necessarily at the same time. A CSSN can be seen as a form of pre-coordinated guide for ST assignments to new chemical concepts—with the caveat that the CSSN is “bootstrapped” from the existing ST assignments—which were post-coordinated. In this capacity, it can streamline the type assignment process and help prevent errors (such as the inconsistent ST assignments that we have previously reported on [21,22,27,30,31]). For example, with a CSSN, a chemical concept must be assigned exactly one existing IST, taking into account the type’s semantic combination defined by its constituent STs. The new concept can be compared to the concepts in the extent of the proposed IST to confirm that they are semantically similar and should be grouped together.

There are times when the creation of a new IST is warranted. For example, new chemical concepts that increase the coverage of the META or offer finer granularity may be justifications. In such cases, the newly added concepts elaborate semantics that were not previously in the UMLS and are more accurately represented with a new IST. Therefore, the new IST can be allowed, for a time, even though its extent size is below the threshold value. After ST assignments are made, it will be seen whether the extent of the new IST has grown enough to validate its existence. If not, those concepts that were temporarily assigned it would be automatically re-assigned an existing IST using one of the techniques described in Section Type coverage for concepts in ISTs falling below the threshold value.

The initial study with the sample of 200 ChEBI compounds showed the feasibility of using a CSSN as an upper-level network for ChEBI. Of course, more studies are necessary to evaluate the effort required for assigning PSTs and ISTs to all ChEBI compounds. The CSSN type assignments would greatly facilitate the assignment of STs to new concepts if the ChEBI were to be integrated into the UMLS.

The main advantage of categorizing the compounds of ChEBI with CSSN types rather than with SN types is a more accurate categorization. For example, if assigned SN types we see five of the 200 ChEBI compounds reviewed would be assigned the ST **Carbohydrate** and ten would be assigned the ST **Lipid**. However, using the CSSN types for categorization, two compounds, alpha-D-galactosyl undecaprenyl diphosphate and galactosylceramide sulfates, are assigned IST **Carbohydrate Lipid**, three are just PST **Carbohydrate**, and eight are just PST **Lipid**. This is a more refined categorization, which also avoids repeated counting of the same concept due to its multiple ST assignments. Similarly, there are 12 compounds in the sample that would be assigned ST **Inorganic Chemical**. But using CSSN categories we see that three of the 12 also have a functional perspective given. With the functional perspective the three ISTs **Inorganic Biomedical or Dental Material**, **Pharmacologic Inorganic Chemical**, and **Hazardous or Poisonous Inorganic Chemical** are each assigned a compound, while the remaining nine are purely categorized with the **Inorganic Chemical** type (see Table 4), thereby maintaining the semantic uniformity of the type categorizations.

There is an inherent conflict between detailed coverage of various areas in biomedicine and the need for a compact semantic network that effectively abstracts it. For applications in specific areas such as genomics, anatomy, chemistry, zoology, physiology, pharmacology, and pathology, there is a need for more detailed categories (types). However, if all such categories were added to the SN, its size would be an order of magnitude higher, and it would lose some of its effectiveness. A possible solution would be to leave the SN with approximately the same size and structurally modify it in other ways, such as converting it into a directed acyclic graph from its current two-tree configuration [23,24]. Alongside the SN, specialty semantic networks for specific areas having enhanced coverage within the appropriate subnetworks could be derived. Those specialty abstraction networks could grow to a size on the same order of magnitude as the SN itself. In this way, effective compact coverage could be achieved for various areas.

In this paper, we have described one methodology based on existing ISTs from the RSN that is applicable to chemicals—as we have demonstrated. Another field where a specialty semantic network can be applicable is for the semantic group of disorders [26,36] where there are meaningfully large intersections. Examples of intersections with extents beyond 1,000 concepts for the disorder domain include: **Anatomical Abnormality** ∩ **Disease or SyndromeCongenital Abnormality** ∩ **Disease or Syndrome**, and **Neoplastic Process** ∩ **Experimental Model of Disease**. For other fields, specialty networks may be derived using other techniques. See, e.g., suggested coverage for genomics [17] building on earlier work in [19].

There exist other abstraction networks for chemistry and life science, which are intended as ontologies that support mapping and integration between the ontologies in OBO. In [37], an upper-level ontology for Chemistry was created based on ChEBI. The foundational relations of ChEBI were analyzed and rewritten to be compatible with the OBO Relation Ontology (RO) [38]. The identified top-level classes of ChEBI were aligned with the Basic Formal Ontology (BFO) types [39]. The other upper domain ontology for the life sciences, BIOTOP [40] was developed from the GENIA ontology [41] (see http://www-tsujii.is.s.u-tokyo.ac.jp/GENIA/home/wiki.cgi?page=GENIA+Ontology). However, in BIOTOP the structure of the GENIA was redesigned to overcome the existing shortcomings. New classes and relation types were introduced into BIOTOP and some classes of the GENIA were removed. The ontologies in the OBO can be integrated by using BIOTOP as a common top level ontology.

Compared to the above described abstract networks, the CSSN can be automatically generated from the UMLS Semantic Network and Metathesaurus. It is better suited for mapping between ChEBI and UMLS source vocabularies. For 200 sample compounds of ChEBI, 96% of were associated with semantic types in the CSSN. According to the results for this sample, it seems that this level of coverage applies for the whole ChEBI.

Both the upper-level ontology for chemistry and BIOTOP include categories such as “Atom” and “Molecule” which are intended for use as top-level ontologies that connect domain ontologies to BFO or as a bridge to integrate different ontologies of OBO. In contrast, the CSSN provides an abstraction network with categories such as “Organic Chemical” and “Hazardous or Poisonous Organic Chemical”, which fit for mapping the ChEBI ontology to UMLS source vocabularies.

## Conclusion

We have presented a methodology for deriving a specialty semantic network for the chemical domain directly from the existing UMLS Semantic Network and Metathesaurus, based on a threshold value that regulated the inclusion of new chemical types. Our CSSN afforded a more refined categorization of the UMLS’s chemical concepts. Such an approach can be repeated for other subdomains of biomedicine such as disorders, although in that domain there are fewer intersections of STs. An important aspect of this work was the fact that it was done without extending the SN itself into a network that is too large. We demonstrated that a CSSN can be derived according to the needs of an application as expressed by the threshold value. At the same time, deriving such a network, rather than building it from scratch, has the benefit of leveraging the wealth of knowledge accumulated in the UMLS over the years.

Among the benefits of a CSSN is the potential for serving as an upper-level semantic network for an ontology of chemicals such as OBO’s ChEBI, providing the chemical ontology with a network playing the same role that the SN does for the META. A CSSN could be a useful guide for the assignment of STs to new concepts as they are added to the UMLS. Generation of a CSSN using a low threshold value yields a useful tool for auditing the META. A CSSN could also be applied during the mapping or alignment between a UMLS source vocabulary and another source of chemical concepts.

## Endnotes

^a^Additional file 2 contains a glossary of acronyms used.

^b^Semantic types are written in bold.

^c^Concepts are written with the preferred term in italics and the Concept Unique Identifier (CUI) following in parenthesis.

## Competing interests

The authors declare that they have no competing interest.

## Authors’ contributions

CPM has written the manuscript, applied and refined the method, and collected data. YP has written the manuscript, designed the method, and directed the research. MH and HG have written the manuscript and designed the method. LC has written the manuscript, identified appropriate type names, and categorized the sample ChEBI compounds. All authors have read and approved the final manuscript.

## Supplementary Material

Additional file 1 A Chemical Specialty Semantic Network with a type threshold value of 300 concepts for UMLS 2009AA. Click here for file

Additional file 2 Glossary of Acronyms. Click here for file
