# Supplementary material for: A chemical specialty semantic network for the Unified Medical Language System
Source: J Cheminform. 2012 May 11;4:9. doi: 10.1186/1758-2946-4-9 (PMC3428652; doi:10.1186/1758-2946-4-9)
Supplement: Additional file 1 — A Chemical Specialty Semantic Network with a type threshold value of 300 concepts for UMLS 2009AA. [file 1758-2946-4-9-S1.doc]

Appendix A

A Chemical Specialty Semantic Network with a type threshold value of 300 concepts for UMLS 2009AA

| **Refined Semantic Type** | **# Concepts** | **Name in CSSN (hierarchically indented)** | | | | | | |
| --- | --- | --- | --- | --- | --- | --- | --- | --- |
| Chemical | 26 | | Chemical | | | | | |
| Chemical Viewed Structurally | 248 | |  | Chemical Viewed Structurally | | | | |
| Organic Chemical | 58347 | |  |  | Organic Chemical | | | |
| Organic Chemical ∩ Pharmacologic Substance | 76832 | |  |  |  | *Pharmacologic Organic Chemical | | |
| Organic Chemical ∩ Pharmacologic Substance ∩ Hazardous or Poisonous Substance | 357 | |  |  |  |  | *Pharmacologic Hazardous or Poisonous Organic Chemical | |
| Organic Chemical ∩ Biologically Active Substance | 4151 | |  |  |  | *Biologically Active Organic Chemical | | |
| Organic Chemical ∩ Hazardous or Poisonous Substance | 2712 | |  |  |  | *Hazardous or Poisonous Organic Chemical | | |
| Organic Chemical ∩ Biomedical or Dental Material | 755 | |  |  |  | *Biomedical or Dental Organic Chemical | | |
| Nucleic Acid, Nucleoside, or Nucleotide | 4731 | |  |  |  | Nucleic Acid, Nucleoside, or Nucleotide | | |
| Nucleic Acid, Nucleoside, or Nucleotide ∩ Pharmacologic Substance | 2275 | |  |  |  |  | *Pharmacologic Nucleic Acid, Nucleoside, or Nucleotide | |
| Nucleic Acid, Nucleoside, or Nucleotide ∩ Biologically Active Substance | 1203 | |  |  |  |  | *Biologically Active Nucleic Acid, Nucleoside, or Nucleotide | |
| Organophosphorus Compound | 919 | |  |  |  | Organophosphorus Compound | | |
| Organophosphorus Compound ∩ Pharmacologic Substance | 923 | |  |  |  |  | *Pharmacologic Organophosphorus Compound | |
| Amino Acid, Peptide, or Protein | 16973 | |  |  |  | Amino Acid, Peptide, or Protein | | |
| Amino Acid, Peptide, or Protein ∩ Biologically Active Substance | 43877 | |  |  |  |  | *Biologically Active Amino Acid, Peptide, or Protein | |
| Amino Acid, Peptide, or Protein ∩ Pharmacologic Substance ∩ Biologically Active Substance | 460 | |  |  |  |  |  | **Pharmacologic Biologically Active Amino Acid, Peptide, or Protein |
| Amino Acid, Peptide, or Protein ∩ Pharmacologic Substance | 6535 | |  |  |  |  | *Pharmacologic Amino Acid, Peptide, or Protein | |
| Amino Acid, Peptide, or Protein ∩ Pharmacologic Substance ∩ Immunologic Factor | 1940 | |  |  |  |  |  | **Pharmacologic Immunologic Amino Acid, Peptide, or Protein |
| Amino Acid, Peptide, or Protein ∩ Indicator, Reagent, or Diagnostic Aid | 462 | |  |  |  |  | *Indicator, Reagent, or Diagnostic Aid Amino Acid, Peptide, or Protein | |
| Amino Acid, Peptide, or Protein ∩ Neuroreactive Substance or Biogenic Amine | 341 | |  |  |  |  | *Neuroreactive or Biogenic Amine Amino Acid, Peptide, or Protein | |
| Amino Acid, Peptide, or Protein ∩ Hazardous or Poisonous Substance | 311 | |  |  |  |  | *Hazardous or Poisonous Amino Acid, Peptide, or Protein | |
| Carbohydrate | 6079 | |  |  |  | Carbohydrate | | |
| Carbohydrate ∩ Pharmacologic Substance | 1894 | |  |  |  |  | *Pharmacologic Carbohydrate | |
| Carbohydrate ∩ Biologically Active Substance | 478 | |  |  |  |  | *Biologically Active Carbohydrate | |
| Amino Acid, Peptide, or Protein ∩ Carbohydrate | 326 | |  |  |  |  | *Amino Acid, Peptide, or Protein Carbohydrate | |
| Lipid | 3514 | |  |  |  | Lipid | | |
| Lipid ∩ Pharmacologic Substance | 1385 | |  |  |  |  | *Pharmacologic Lipid | |
| Lipid ∩ Biologically Active Substance | 448 | |  |  |  |  | *Biologically Active Lipid | |
| Steroid | 4883 | |  |  |  |  | Steroid | |
| Steroid ∩ Pharmacologic Substance | 2980 | |  |  |  |  |  | *Pharmacologic Steroid |
| Eicosanoid | 537 | |  |  |  |  | Eicosanoid | |
| Eicosanoid ∩ Pharmacologic Substance | 436 | |  |  |  |  |  | *Pharmacologic Eicosanoid |
| Inorganic Chemical | 2493 | |  |  | Inorganic Chemical | | | |
| Pharmacologic Substance ∩ Inorganic Chemical | 1983 | |  |  |  | *Pharmacologic Inorganic Chemical | | |
| Element, Ion, or Isotope | 1004 | |  |  | Element, Ion, or Isotope | | | |
| Chemical Viewed Functionally | 174 | |  | Chemical Viewed Functionally | | | | |
| Pharmacologic Substance | 14181 | |  |  | Pharmacologic Substance | | | |
| Antibiotic | 546 | |  |  |  | Antibiotic | | |
| Organic Chemical ∩ Antibiotic | 3071 | |  |  |  |  | *Organic Antibiotic | |
| Amino Acid, Peptide, or Protein ∩ Antibiotic | 490 | |  |  |  |  | *Amino Acid, Peptide, or Protein Antibiotic | |
| Biomedical or Dental Material | 3411 | |  |  | Biomedical or Dental Material | | | |
| Biomedical or Dental Material ∩ Inorganic Chemical | 300 | |  |  |  | *Inorganic Biomedical or Dental Material | | |
| Biologically Active Substance | 1026 | |  |  | Biologically Active Substance | | | |
| Pharmacologic Substance ∩ Biologically Active Substance | 716 | |  |  |  | *Pharmacologic Biologically Active Substance | | |
| Organic Chemical ∩ Pharmacologic Substance ∩ Biologically Active Substance | 507 | |  |  |  |  | **Pharmacologic Biologically Active Organic Chemical | |
| Biologically Active Substance ∩ Hazardous or Poisonous Substance | 490 | |  |  |  | *Hazardous or Poisonous Biologically Active Substance | | |
| Neuroreactive Substance or Biogenic Amine | 20 | |  |  |  | Neuroreactive Substance or Biogenic Amine | | |
| Hormone | 147 | |  |  |  | Hormone | | |
| Steroid ∩ Pharmacologic Substance ∩ Hormone | 1051 | |  |  |  |  | *Pharmacologic Steroid Hormone | |
| Amino Acid, Peptide, or Protein ∩ Hormone | 426 | |  |  |  |  | *Amino Acid, Peptide, or Protein Hormone | |
| Amino Acid, Peptide, or Protein ∩ Pharmacologic Substance ∩ Hormone | 575 | |  |  |  |  |  | **Pharmacologic Amino Acid, Peptide, or Protein Hormone |
| Enzyme | 233 | |  |  |  | Enzyme | | |
| Amino Acid, Peptide, or Protein ∩ Enzyme | 24229 | |  |  |  |  | *Amino Acid, Peptide, or Protein Enzyme | |
| Amino Acid, Peptide, or Protein ∩ Pharmacologic Substance ∩ Enzyme | 492 | |  |  |  |  |  | **Pharmacologic Amino Acid, Peptide, or Protein Enzyme |
| Vitamin | 117 | |  |  |  | Vitamin | | |
| Pharmacologic Substance ∩ Vitamin | 519 | |  |  |  |  | *Pharmacologic Vitamin | |
| Organic Chemical ∩ Pharmacologic Substance ∩ Vitamin | 1331 | |  |  |  |  |  | **Pharmacologic Organic Vitamin |
| Immunologic Factor | 7181 | |  |  |  | Immunologic Factor | | |
| Amino Acid, Peptide, or Protein ∩ Immunologic Factor | 12662 | |  |  |  |  | *Immunologic Amino Acid, Peptide, or Protein | |
| Pharmacologic Substance ∩ Immunologic Factor | 1065 | |  |  |  |  | *Pharmacologic Immunologic Factor | |
| Receptor | 125 | |  |  |  | Receptor | | |
| Amino Acid, Peptide, or Protein ∩ Receptor | 3783 | |  |  |  |  | *Amino Acid, Peptide, or Protein Receptor | |
| Indicator, Reagent, or Diagnostic Aid | 4350 | |  |  | Indicator, Reagent, or Diagnostic Aid | | | |
| Organic Chemical ∩ Indicator, Reagent, or Diagnostic Aid | 4624 | |  |  |  | *Organic Indicator, Reagent, or Diagnostic Aid | | |
| Indicator, Reagent, or Diagnostic Aid ∩ Inorganic Chemical | 544 | |  |  |  | *Inorganic Indicator, Reagent, or Diagnostic Aid | | |
| Pharmacologic Substance ∩ Indicator, Reagent, or Diagnostic Aid | 482 | |  |  |  | *Pharmacologic Indicator, Reagent, or Diagnostic Aid | | |
| Organic Chemical ∩ Pharmacologic Substance ∩ Indicator, Reagent, or Diagnostic Aid | 337 | |  |  |  |  | **Pharmacologic Organic Indicator, Reagent, or Diagnostic Aid | |
| Hazardous or Poisonous Substance | 430 | |  |  | Hazardous or Poisonous Substance | | | |
