# Supplementary material for: A chemical specialty semantic network for the Unified Medical Language System
Source: J Cheminform. 2012 May 11;4:9. doi: 10.1186/1758-2946-4-9 (PMC3428652; doi:10.1186/1758-2946-4-9)
Supplement: Additional file 2 — Glossary of Acronyms. [file 1758-2946-4-9-S2.doc]

Appendix B

Glossary of Acronyms

BFO: Basic Formal Ontology – A top-level ontology composed of a series of sub-ontologies. (See http://www.ifomis.org/bfo)

BIOTOP: A top-domain ontology that provides definitions for the foundational entities of biomedicine. (See http://www.imbi.uni-freiburg.de/ontology/biotop)

ChEBI: Chemical Entities of Biological Interest – An ontology of molecular entities that are either products of nature or synthetic products used to intervene in the processes of living organisms. (See http://www.ebi.ac.uk/chebi)

CSSN: Chemical Specialty Semantic Network – A semantic network that is derived from the assignments of STs to concepts in the META and offers both improved semantic uniformity and control over granularity.

IST: Intersection Semantic Type – A type of an RSN that is derived from UMLS concepts that have assignments to multiple STs.

META: The UMLS Metathesaurus – A knowledge source of the UMLS that is composed of about 150 integrated source vocabularies.

OBO: The Open Biological and Biomedical Ontologies Foundry – A suite of orthogonal interoperable reference ontologies in the biomedical domain. (See http://obofoundry.org)

PST: Pure Semantic Type – A semantic type of an RSN that is derived from UMLS concepts that have assignments to only one ST.

RO: OBO Relations Ontology – An ontology of core relations used by OBO ontologies. (See http://obofoundry.org/ro)

RSN: Refined Semantic Network – A semantic network that is derived from the assignments of STs to concepts in the META and offers improved semantic uniformity.

RST: Refined Semantic Type – Either a PST or an IST of the RSN.

SN: UMLS Semantic Network – A knowledge source of the UMLS that categorizes all concepts of the META.

ST: Semantic Type – A category of the SN.

UMLS: Unified Medical Language System -- A set of files and software that brings together many health and biomedical vocabularies and standards to enable interoperability between computer systems. (See http://www.nlm.nih.gov/research/umls/quickstart.html)
